# Manufacturing and characterisation of 3D-printed sustained-release Timolol implants for glaucoma treatment

**DOI:** 10.1007/s13346-024-01589-8

**Published:** 2024-04-05

**Authors:** Fathima Paleel, Mengqi Qin, Aristides D. Tagalakis, Cynthia Yu-Wai-Man, Dimitrios A. Lamprou

**Affiliations:** 1 School of Pharmacy, Queen’s University Belfast, BT9 7BL Belfast, UK; 2 Faculty of Life Sciences & Medicine, King’s College London, SE1 7EH London, UK; 3 Department of Biology, Edge Hill University, L39 4QP Ormskirk, UK

**Keywords:** Glaucoma, Timolol, 3D printing, Drug-eluting implants, Sustained-release

## Abstract

Timolol maleate (TML) is a beta-blocker drug that is commonly used to lower the intraocular pressure in glaucoma. This study focused on using a 3D printing (3DP) method for the manufacturing of an ocular, implantable, sustained-release drug delivery system (DDS). Polycaprolactone (PCL), and PCL with 5 or 10% TML implants were manufactured using a one-step 3DP process. Their physicochemical characteristics were analysed using light microscopy, scanning electronic microscopy (SEM), differential scanning calorimetry (DSC) / thermal gravimetric analysis (TGA), and Fourier-transform infrared spectroscopy (FTIR). The in vitro drug release was evaluated by UV-spectrophotometry. Finally, the effect of the implants on cell viability in human trabecular meshwork cells was assessed. All the implants showed a smooth surface. Thermal analysis demonstrated that the implants remained thermally stable at the temperatures used for the printing, and FTIR studies showed that there were no significant interactions between PCL and TML. Both concentrations (5 & 10%) of TML achieved sustained release from the implants over the 8-week study period. All implants were non-cytotoxic to human trabecular cells. This study shows proof of concept that 3DP can be used to print biocompatible and personalised ocular implantable sustained-release DDSs for the treatment of glaucoma.

**Graphical Abstract:**

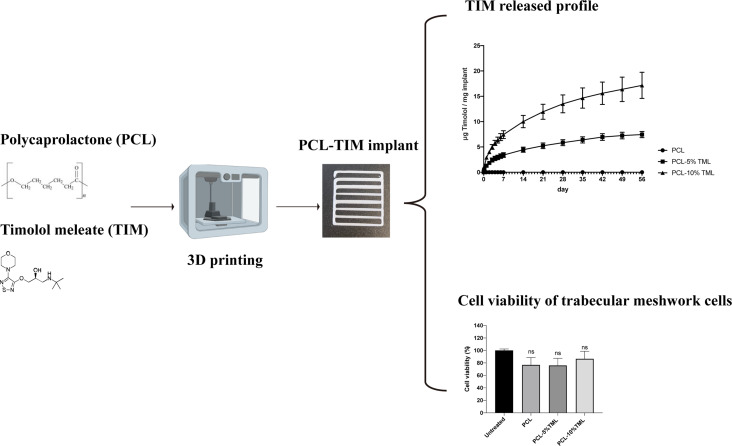

## Introduction

Glaucoma is the main cause of irreversible blindness worldwide [1]. Considering that there were 76 million affected people in 2020, and this value is estimated to reach 111.8 million by 2040 [2, 3], effective therapeutic interventions have become an urgent imperative to mitigate the global burden of this disease. There are various pharmacological treatment options to manage glaucoma, including beta-blockers, prostaglandin analogues, alpha-agonists, and topical carbonic anhydrase [4]. One of the first-line therapeutic agents in glaucoma management is timolol (TML), a non-selective beta-blocker known for its ability to reduce aqueous humour production and to lower the intraocular pressure (IOP) [5, 6]. This medication is available in various formulations, including eye drops and gel solutions, which have been widely used in clinical practice [7]. Despite its proven efficacy, the limitations of conventional delivery methods have sparked interest in developing sustained implantable drug delivery systems (DDSs) to enhance the bioavailability and long-term therapeutic effects of anti-glaucoma drugs [8].

Traditional treatments for glaucoma, such as eye drops and oral medications, have shown promising results in managing IOP; however, issues such as poor patient compliance, low bioavailability, and the daily treatment burden by eye drops underscore the need for sustained DDSs to optimise glaucoma management [9]. To address these problems, multiple ocular DDSs with the aim to deliver therapeutic levels of anti-glaucoma drugs in the eye for extended periods have emerged. Durysta™ (Allergan), a rod-shaped intracameral implant that can continuously release bimatoprost over 4 to 6 months, is the first Food and Drug Administration (FDA) approved drug implant for glaucoma management [10]. In addition, there are some sustained-release prostaglandin-eluting implants that are currently in the late clinical development and are considered as promising alternatives to daily eye drops, such as Glaukos’ iDose™, a travoprost-containing implant which is designed to release travoprost in the anterior chamber for 6 months [9], and OTX-TIC, an intracameral hydrogel-based implant which can release travoprost for up to 4 months [11]. Apart from glaucoma, sustained-release implants also stand as a pioneering breakthrough for the treatment of other ophthalmic disorders. Ozurdex®, a dexamethasone intravitreal implant, has been widely used in the treatment of uveitis or macular oedema by sustainably releasing dexamethasone for up to 6 months [12].

3D printing (3DP), also termed additive manufacturing (AM), is using a Computer-Aided Design (CAD) for the manufacturing of 3D objects layer-by-layer (LbL). 3D printing’s feasibility in medical applications lies in its precision and customization, enabling the creation of accurate, patient-specific implants. Advanced CAD technology and real-time monitoring ensure strict quality control [13]. The inherently sterile environment and the use of sterile materials make it suitable for medical implant production, minimizing infection risks. While initial setup costs exist, long-term cost-effectiveness is achieved by reducing the need for extensive tooling and molds[14]. 3DP is increasingly used to make DDSs and is being researched in a range of different diseases as it allows for localised drug administration over a sustained period of time [15, 16]. Recently, 3DP technologies have also been developed in ophthalmology. For example, an anti-fibrotic drug, 5-Fluorouracil (5-FU), has successfully been loaded into a rod-like Polycaprolactone (PCL)-chitosan implant using 3DP method, with the aim to prevent conjunctival fibrosis after glaucoma filtration surgery [17]. Mohamdeen and coworkers successfully developed the personalised ocular contact lenses loaded with TML by 3DP technology for the treatment of glaucoma, providing a sustained release for 7 days [18], however, the contact lenses suffer from challenges including low bioavailability caused by drug permeation and irritation of the cornea. In this research, the fused deposition modeling (FDM) technique, one of the most common 3DP technology, in a one-step process, was used. This process involves several key steps such as: initial scaffold design, preparation of raw materials, extrusion of the scaffold filament at a predetermined temperature, and cooling and solidification of the extrudates. Notably, this one-step approach streamlines the manufacturing process, enhancing efficiency and ensuring consistent interface properties of the implant in comparison to multi-step 3DP processes [19].

Here, to achieve a reduced TML loss and extend drug retention, we developed two types of 3DP intracameral implants capable of releasing TML over 8 weeks. In this work, PCL implants were manufactured by a one-step 3DP process. The integration of PCL, a biocompatible and biodegradable polymer, with two different percentages of TML represents a promising synergy that not only ensures sustained drug release but also exhibits good biocompatibility and safety. It is demonstrated that the one-step 3D printing process provides an advantage for personalised medication, and development of patient-specific ocular implants, which can deliver sustained doses of TML directly to the affected site, circumventing the challenges posed by conventional daily eye drops and the reliance on patient compliance. This study also acts as a proof of concept, building upon existing research on 3D printing for ocular use. 5% and10% concentrations of Timolol were used as there is a lack of research within DDS using concentrations higher than 5%. This study further established the changes or lack of changes in the printing process, and subsequently the outcome of the implant with a much higher drug dosage than most found in the existing literature.

## Materials and methods

### Materials

Polycaprolactone powder (PCL; MW 50,000 Daltons; Tm = 58 °C) was purchased from Polysciences Inc. PCL with 50,000 Daltons molecular weight was used following the known suitable viscosity of the melting polymer needed for 3DP from previous studies [17, 20]. Timolol maleate (TML) and phosphate-buffered saline (PBS, pH 7.4) were purchased from Sigma-Aldrich. Dulbecco’s modified Eagle’s medium (DMEM) and foetal calf serum were purchased from Thermo Scientific (Gibco, UK). Penicillin and streptomycin were purchased from Sigma-Aldrich. CellTiter 96® AQueous One Solution Reagent was purchased from Promega (Southampton, UK).

### Synthesis of Timolol-loaded Implant by 3D printed process

**Fig. 1:**
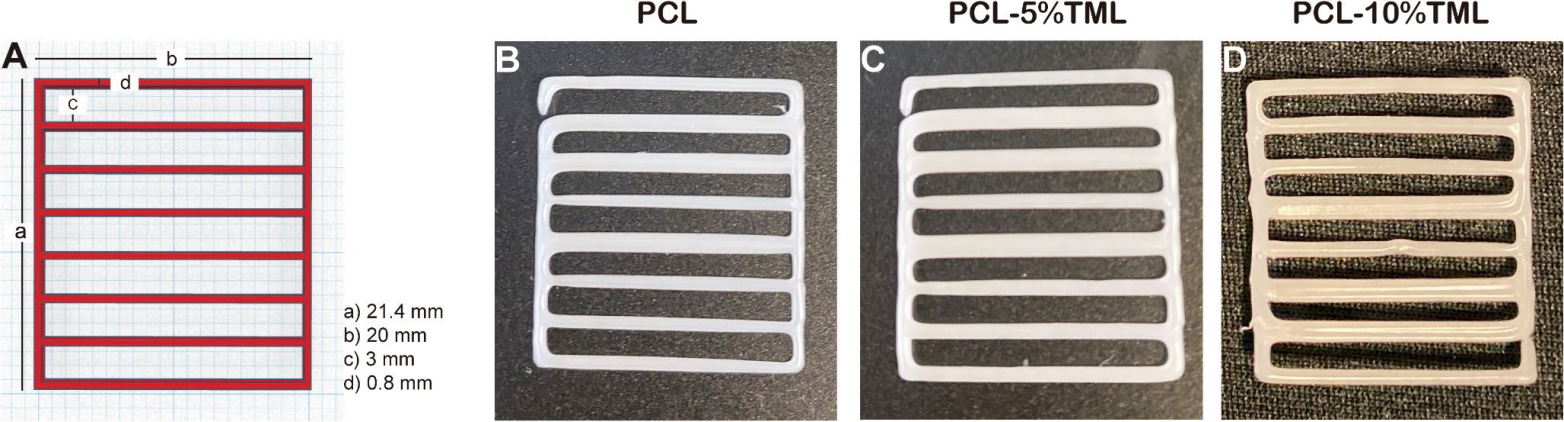
Dimensions of the implant design (**A**) and the final 3D printed structures: PCL (**B**), PCL-5%TML (**C**) and PCL-10%TML (**D**)

Tinkercad, an online 3D CAD design tool (Fig. 1a), was used to design scaffold structures, which were then divided to smaller implantable systems. The Biox^™^ 3D Bioprinter (Cellink, Sweden) with a thermoplastic printhead was employed, using a 22G conical needle (0.41 mm inner diameter) at 1 mm/s speed. PCL only implants were manufactured by adding the powder directly into the thermoplastic printhead without adding any solvent and fill maximum half of the cartridge to ensure the most efficient heating of PCL. Implants containing both PCL and the timolol maleate were added together in thermoplastic cartridge as the powder form and vortexed for 5 min at 60 s intervals. The layer height for printing was set at 0.8 mm and the infill density was set at 0%. Each implant was composed of 2 layers. To achieve good printability, optimisation of the printing pressures and temperatures was required to achieve good flow (data not shown). The PCL extrusion was smooth, and the implant was fully formed at 150 °C and at the pressure of 175 kPa. With the addition of TML, the temperature remained the same; however, the pressure had to be increased to 190 kPa.

### Microscopic evaluation

Light microscopy images of all implants were taken using the Olympus CKX41 inverted microscope with CellSens Standard 1.13 (Build 13,479) software. The surface morphology of the implants was further analysed using a Scanning Electron Microscope (SEM; Hitachi TM3030 SEM, Tokyo, Japan). Images were taken at a magnification of 5000x in the Energy Dispersive X-Ray (EDX) condition, focusing on the successful melting of PCL and the possible presence of TML powder on the implant’s surface.

### Thermal properties

The thermal behaviours of the raw material as well as those of the printed implants were studied using the differential scanning calorimeter (DSC) 214 Polyma (NETZSCH-Geratebau GmbH, Wolverhampton, UK). The samples were weighed and then placed in aluminium crucibles. Starting from 25˚C (Room Temperature, RT) the samples were heated up to 300 °C. Scanning rate was 10 K/min, under protection of nitrogen purge gas (flow rate 20.0 mL/min).

The thermal gravimetric analyser (TGA; Q50, TA, USA) was used to explore the influence of varying concentrations of TML on the thermal stability of 3D-printed implants over a progressive increase of temperature. The samples were weighed and placed in aluminium crucibles. The ramp was set at 20 °C/min from RT to 500 °C, a temperature corresponding to the complete degradation of PCL, under the protection of nitrogen purge gas (flow rate 50.0 mL/min) [20].

### Fourier-transform infrared spectroscopy (FTIR)

Attenuated Total Reflection FTIR (ATR-FTIR) Nicolet^™^ iS50 FTIR Spectrometer (Thermo-Fisher Scientific) was used to analyse both powder and 3DP implants to detect any potential chemical interactions or chemical modifications. Each sample spectrum was obtained between 4000 cm and ^1^ and 400 cm^-1^. The samples were run at a resolution of 4 cm^-1^ with 64 scans after background correction.

### In vitro drug release test and implants weight changes

TML was analysed by UV spectrophotometer for determination of the in vitro release using a modified established protocol in our labs [21]. Briefly, the 3D printed implant was cut into 1 cm, then the implant section’s weight was measured before being immersed in a vial containing 1 mL of PBS (pH = 7.4) and kept in an incubator at 37 °C [21–24]. At each time point (e.g., 1 h, 2 h, 4 h, 6 h, 1 day, 2 days, 3 days, 4 days, 5 days, 6 days, 7 days, 2 weeks, 3 weeks, 4 weeks, 5 weeks, 6 weeks, 7 weeks and 8 weeks), the entire solution was removed and replaced by 1 mL of fresh PBS. Each sample was analysed using a UV-spectrophotometer (Cole-Parmer, Staffordshire, UK) at a wavelength of 297 nm [25]. Samples from all time points were run in triplicates.

The implants were placed in PBS at 37 °C. The initial weight of the implants was measured at day 0. After the drug-releasing experiment, the implants were thoroughly dried, and the weights were determined. The experiment was performed in triplicates.

### In vitro biocompatibility study

Trabecular meshwork tissues from glaucoma patients were used to culture human TM cells [26]. The samples were obtained with the patient’s informed consent and all experimental protocols were approved by the West of Scotland Research Ethics Committee (REC 19/WS/0146). The human TM cells were grown in complete culture media consisting of DMEM, 10% foetal calf serum, and 100 U/mL penicillin/ 0.1 mg/mL streptomycin and cultured in incubators at 37 °C with 5% CO_2_ and 95% humidity. The human TM cells were plated in a 96-well plate at a density of 6.25 × 10^3^ cells per well. The experiment was done in triplicates for each type of implant (PCL, PCL-5%TML, PCL-10%TML) and untreated control cells. The cells were treated with 50 µL complete media and 50 µL media containing the drug solution collected from each implant on day 1, day 3, day 5, day 7, week 2, week 3 and week 4. After 24 h, the cell media was replaced with fresh culture media, followed by the addition of 20 µL of CellTiter 96® AQueous One Solution Reagent. The plate was incubated for 2 h at 37 °C with 5% CO_2_ and 95% humidity. The plate was then read using a plate reader, PHERAstar FS (BMG LABTECH), set at 490 nm absorbance, and the results were normalised against untreated control cells. The experiment was performed in triplicates.

### Statistical analysis

Statistical significance was determined by One-way ANOVA followed by post hoc test. The statistical significance was indicated as follows: *, *p* < 0.05; **, *p* < 0.01; ***, *p* < 0.001; ****, *p* < 0.0001; ns, not significant. All data are expressed as mean ± SD.

## Results

### Implant design and printing

Implants were designed by Tinkercad and manufactured by an extrusion Bioprinting method (Fig. 1). To ensure material homogeneity, the implants containing TML were vortexed for 10 min before being funnelled into the printing syringes. The extrusion temperature was set at 150 °C, which allowed suitable material extrusion in all implant types without causing degradation of PCL. The printing speed was set to 1 mm/s to allow the material to cool before the second layer was added, thus preventing clumpy depositions. Table 1 represents the dimensions of implant design and actual scaffolds.

**Table 1:** Dimensions of the designed implants and the final 3D-printed structures

Dimensions	Implant design (mm)	PCL implant (mm)	PCL + 5% TML implant (mm)	PCL + 10% TML implant (mm)
Width of implant	0.80	1.02 ± 0.41	1.56 ± 0.21	1.40 ± 0.17
Length of implant	20.00	18.88 ± 0.35	20.04 ± 0.28	19.47 ± 0.38
Hollow inner length	3.00	1.83 ± 0.05	1.68 ± 0.14	1.68 ± 0.13

### Surface analysis

**Fig. 2:**
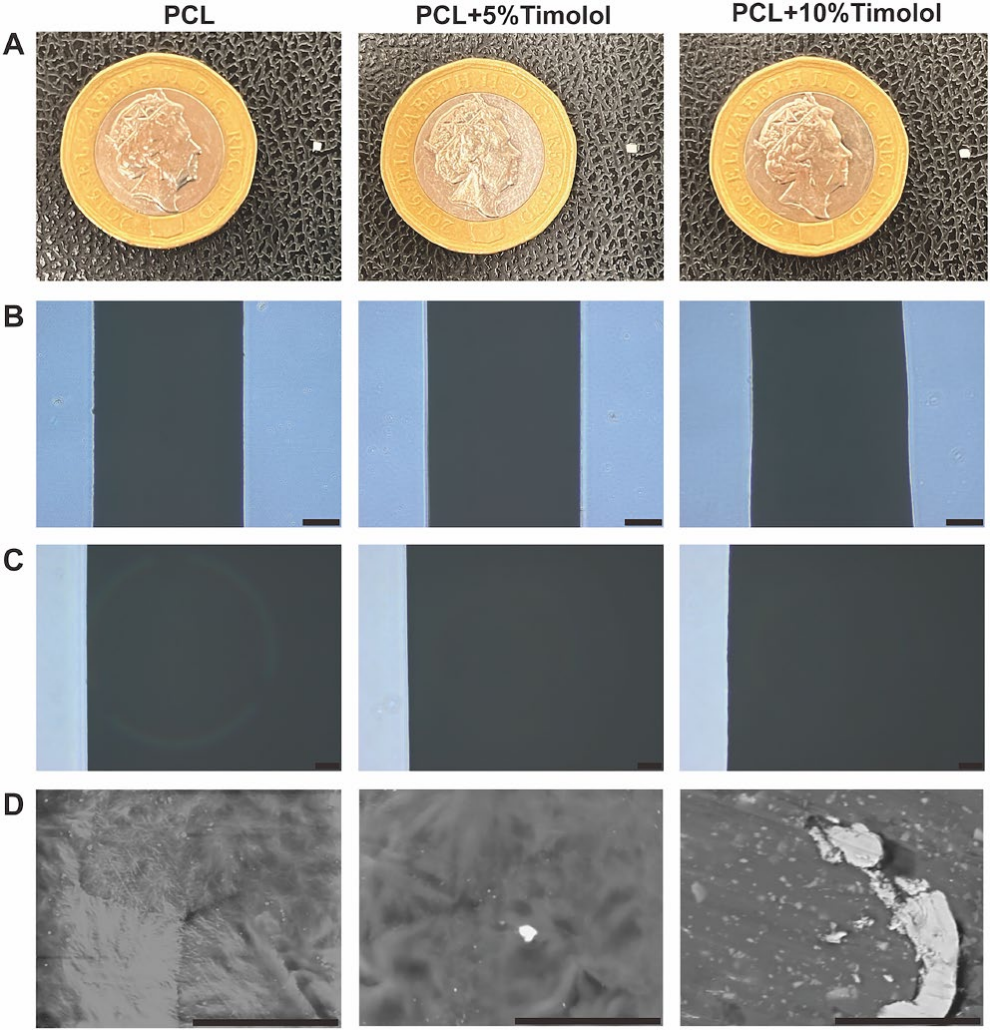
(**A**) Photographs of the PCL, PCL-5%TML and PCL-10%TML implants versus a pound sterling coin for size comparison. Light microscopy images were taken at magnifications of (**B**) 4x, scale bar: 200 μm and (**C**) 10x, scale bar: 50 μm. (**D**) SEM images were taken at magnification of 5000x, scale bar: 20 μm

After their fabrication by 3DP, the implants were sectioned into 1 mm in length, rendering them suitable for intraocular implantation (Fig. 2). All the implants under light microscopy showed consistent optical transmission, indicating that the raw material powders were well mixed, and implants were homogenous in density (Fig. 2B and C). SEM was used to analyse the implant’s surface and to examine the extrusion of the material, observing a smooth surface for all implants. However, for PCL-5%TML and PCL-10%TML implants, the formation of white clumps could be observed, possibly because TML did not melt during the printing process, as its melting temperature is above 200 °C (Fig. 2D).

### Implant thermal behaviour

**Fig. 3:**
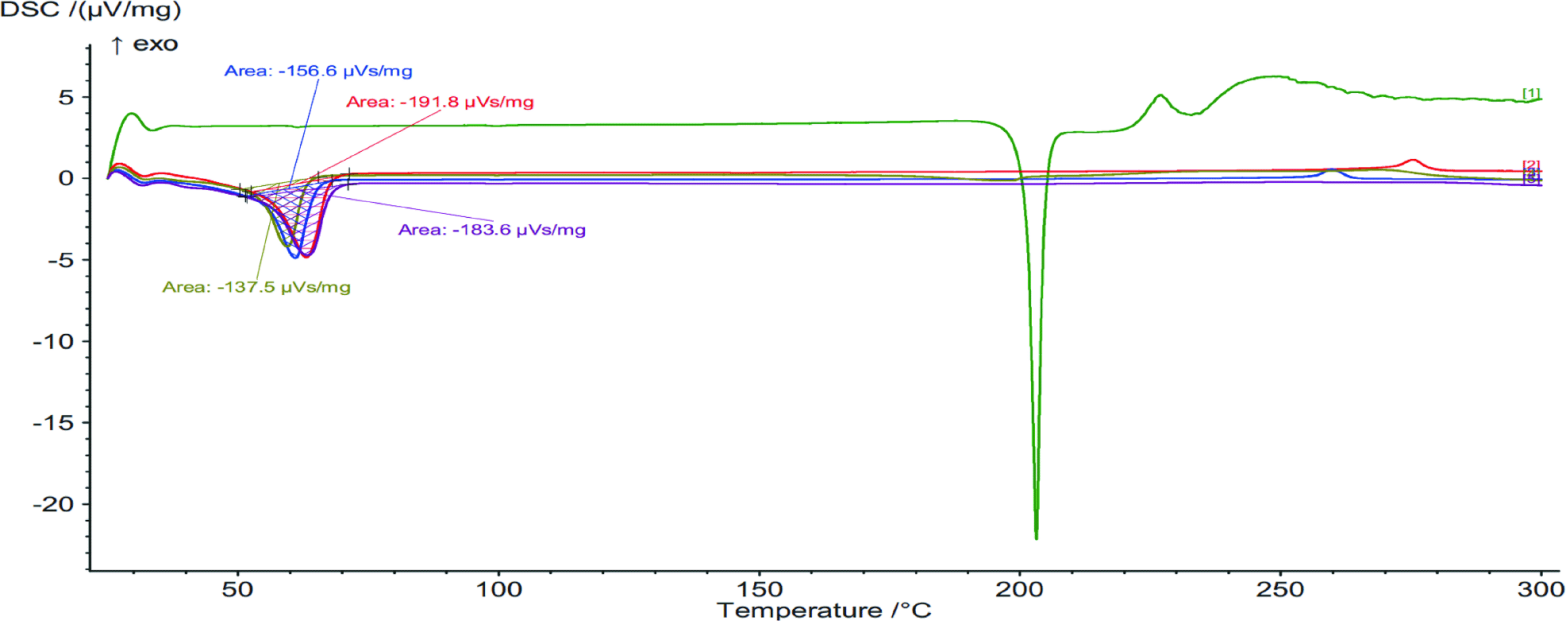
Differential scanning calorimeter (DSC) heating curves of TML powder (green), PCL powder (red), PCL implant (blue), PCL-5%TML implant (purple), and PCL-10%TML implant (army green)

DSC and TGA were used to determine the thermal stability of the implants and powders. The data obtained allow for the evaluation of the printing parameters and to observe any potential effects. The DSC thermogram analysis (Fig. 3; Table 2) encompasses all implants and raw material powders. All implants and materials, except for TML powder, had an onset temperature of around 35 °C to 45 °C. An endothermic peak was also observed in all implants, and for the PCL powder within 59 °C to 63 °C. In comparison to the PCL implant, the endothermic temperatures exhibited a slight change, being 2 degrees higher in the PCL-5%TML implants and 2 degrees lower in the PCL-10%TML implants. The onset temperature of TML is around 200 °C with the endothermic peak at about 204 °C. An exothermic peak can be observed at 230 °C, past TML’s degradation temperature. However, the DSC thermograms did not show any TML-related peaks, possibly due to the conversion of TML to an amorphous state when combined with the polymer [27], similar results have been previously reported not only for TML [18, 27], but also for other types of drugs such as dipyridamole [28]. Nevertheless, it’s crucial to note that the DSC analyse alone may not provide sufficient evidence to conclusively prove the amorphization of TML. Therefore, additional techniques, such as X-ray diffraction, could potentially be employed to comprehensively determine the solid-state characteristics of the drug.

**Table 2:** Raw material and printed implant’s onset temperatures and endothermic peaks obtained from DSC graphical analysis

Material	Onset Temperature (^o^C)	Endothermic Peak (^o^C)
TML powder	196.5	203.2
PCL powder	43.9	63.1
PCL implant	35.1	61.0
PCL-5%TML implant	40.2	63.3
PCL-10%TML implant	44.2	59.5

**Fig. 4:**
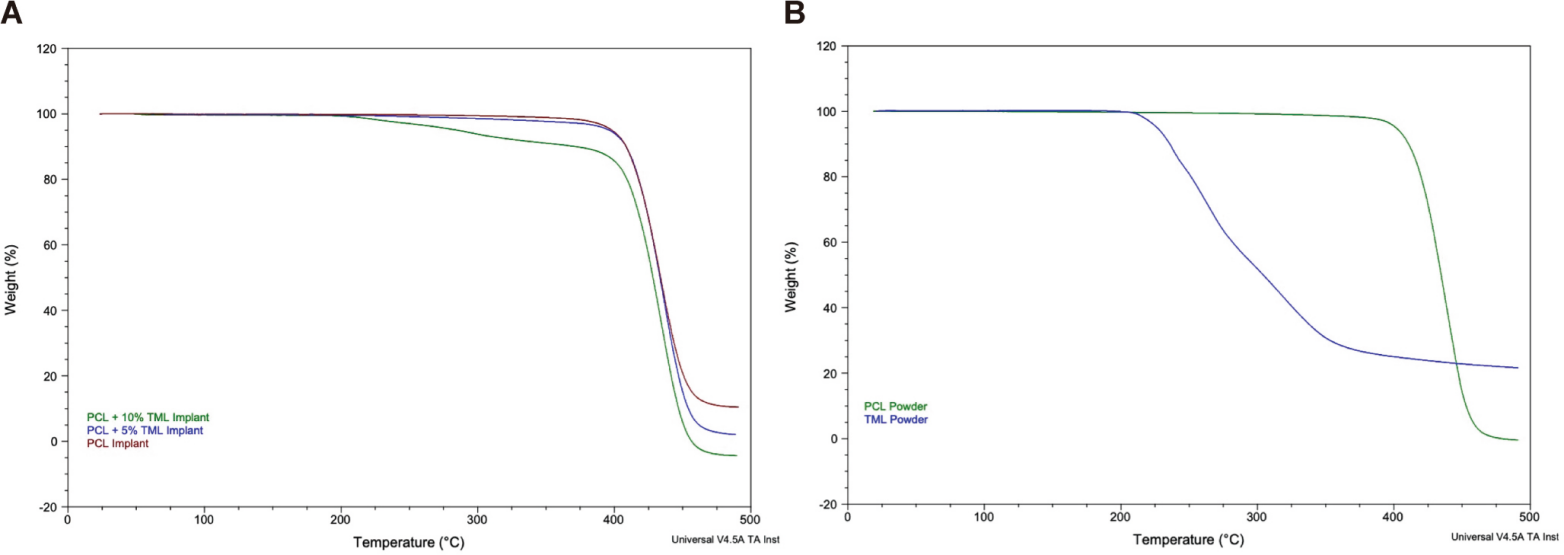
Thermal gravimetric analyser (TGA) results of (**A**) PCL (brown), PCL-5%TML (blue) and PCL-10%TML implants (army green), and (**B**) PCL (green) and TML (blue) powders

To further determine the thermal stabilities, TGA analysis was undertaken to illustrate the material’s behaviour during the printing process. A maximum temperature of 500 °C was selected to fully capture PCL and TML degradation, as well as mixing these materials with affected polymer stability (Fig. 4A). Under the printing temperature of 150 °C, the PCL implant had no weight loss, and was thermally stable until 400 °C. The PCL-5%TML implant showed similar thermal stability with the temperature below 400 °C yet exhibited greater material loss when the temperature reached 400 °C compared to the PCL implant. In contrast, the PCL-10%TML started degrading at around 200 °C, as some weight loss can be observed from this point onwards. These findings underscore that the addition of TML, particularly at a 10% concentration, leads to a reduction in thermal stability in PCL implants. TML powder (Fig. 4B) gradually degrades and loses weight from a temperature of around 200 °C, which is like the implant containing 10% TML concentration.

### Spectroscopic analysis

**Fig. 5:**
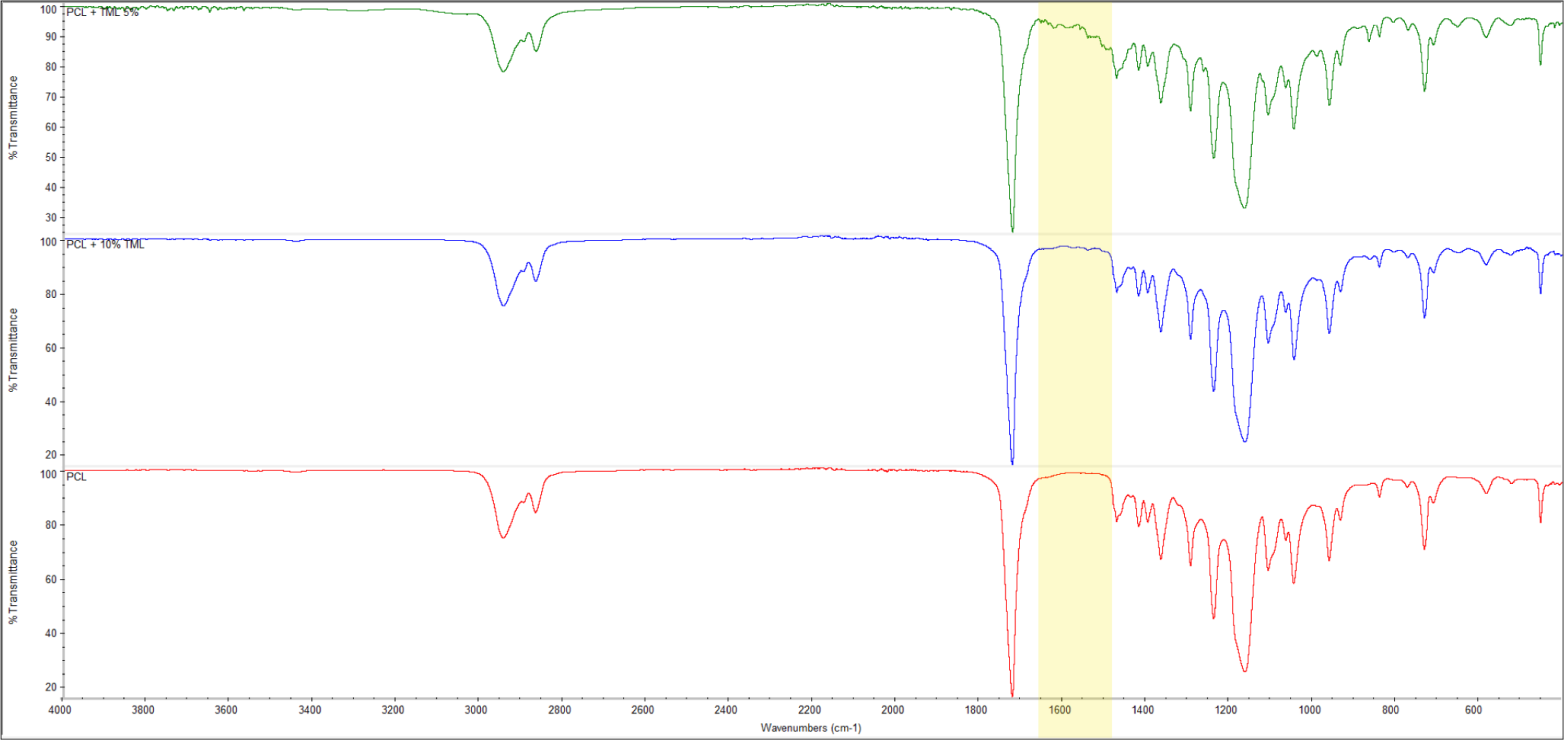
Fourier-transform infrared spectroscopy (FTIR) spectra of the three implant types: PCL (red), PCL-5%TML (army green), PCL-10%TML (blue). The yellow highlighted region shows a difference in spectra between the three implants

FTIR was carried out to identify whether the printing parameters resulted in significant changes in the chemical interactions between PCL and TML (Fig. 5). No significant differences were observed between implants. Most peaks were characteristic of PCL. Previously published studies have reported absorption at 2900 cm^-1^, 2800 cm^-1^, and 1700 cm^-1^ in PCL, which are also seen in Fig. 5 [27]. These absorptions correspond to bonds CH3 (asymmetric stretching), CH3 (symmetric stretching), and C––O (stretching), respectively [29]. Typical TML peaks are observed at 2966 cm^-1^ and 2855 cm^-1^, corresponding with the OH group’s presence. A peak at around 1711 cm^-1^ and 1494 cm^-1^ is due to the NH bond [30]. The 5% drug-loaded implants demonstrate a slightly varied absorption pattern to that of the other implants, at about 1500 cm^-1^ and 1650 cm^-1^, possibly elucidating to the NH group reported in pure TML powder. This could not be seen in the 10% implant due to the minute sample size, thus, the tiny amount used in the study may have contained more TML simply by chance. The difference in chemical composition/absorption is most notable with the 5% TML concentration. Overall, the similarity in the spectra between the control (PCL) implants and those containing TML, indicates a lack of unwanted chemical bond formation between the polymer PCL and TML during the printing process.

### In vitro drug release test and implant weight loss

**Fig. 6:**
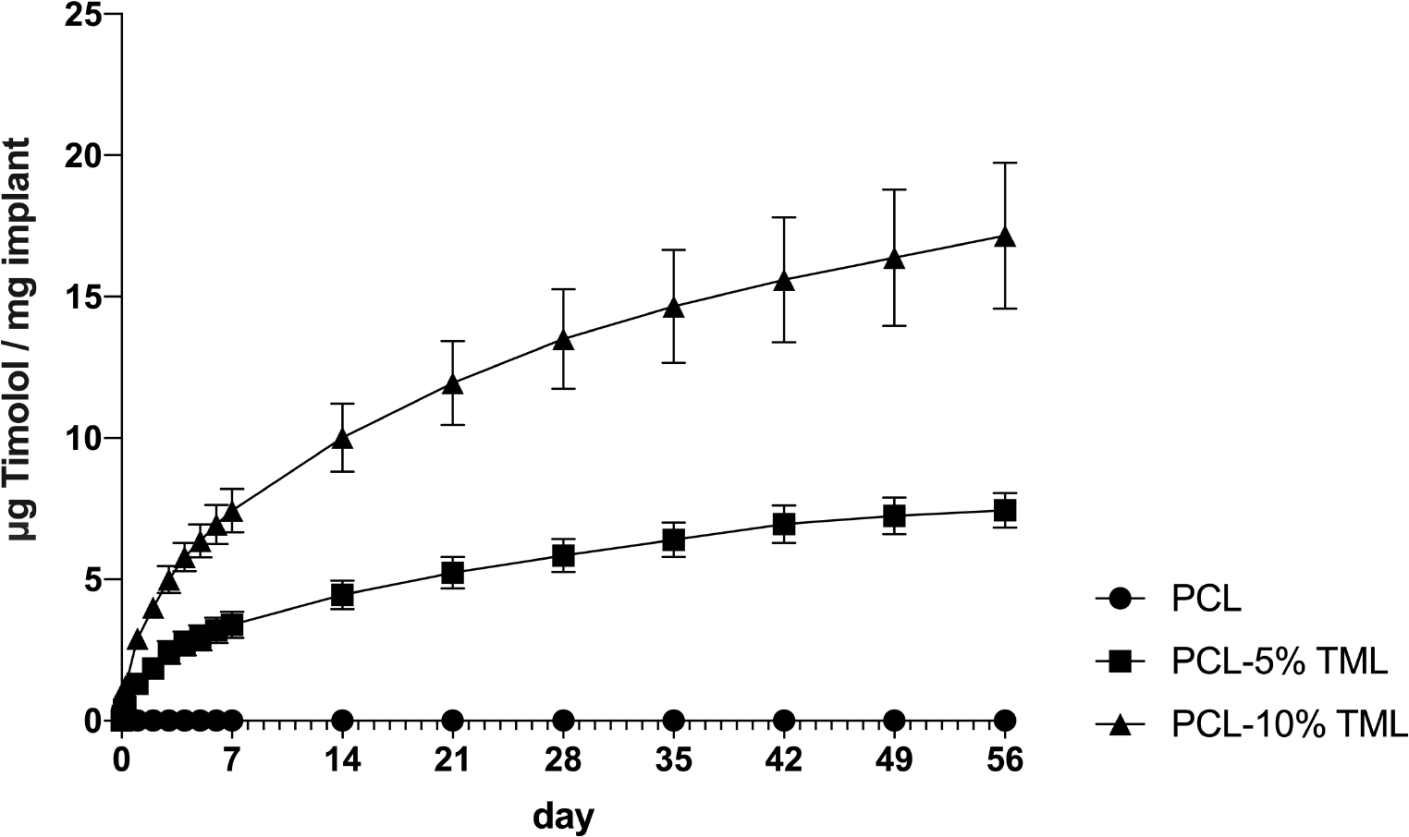
Drug release over 8 weeks measured by UV-spectrophotometer showing the sustained release of TML from the PCL, PCL-5%TML and PCL-10%TML implants. Results represent mean ± SD, *N* = 3

**Fig. 7:**
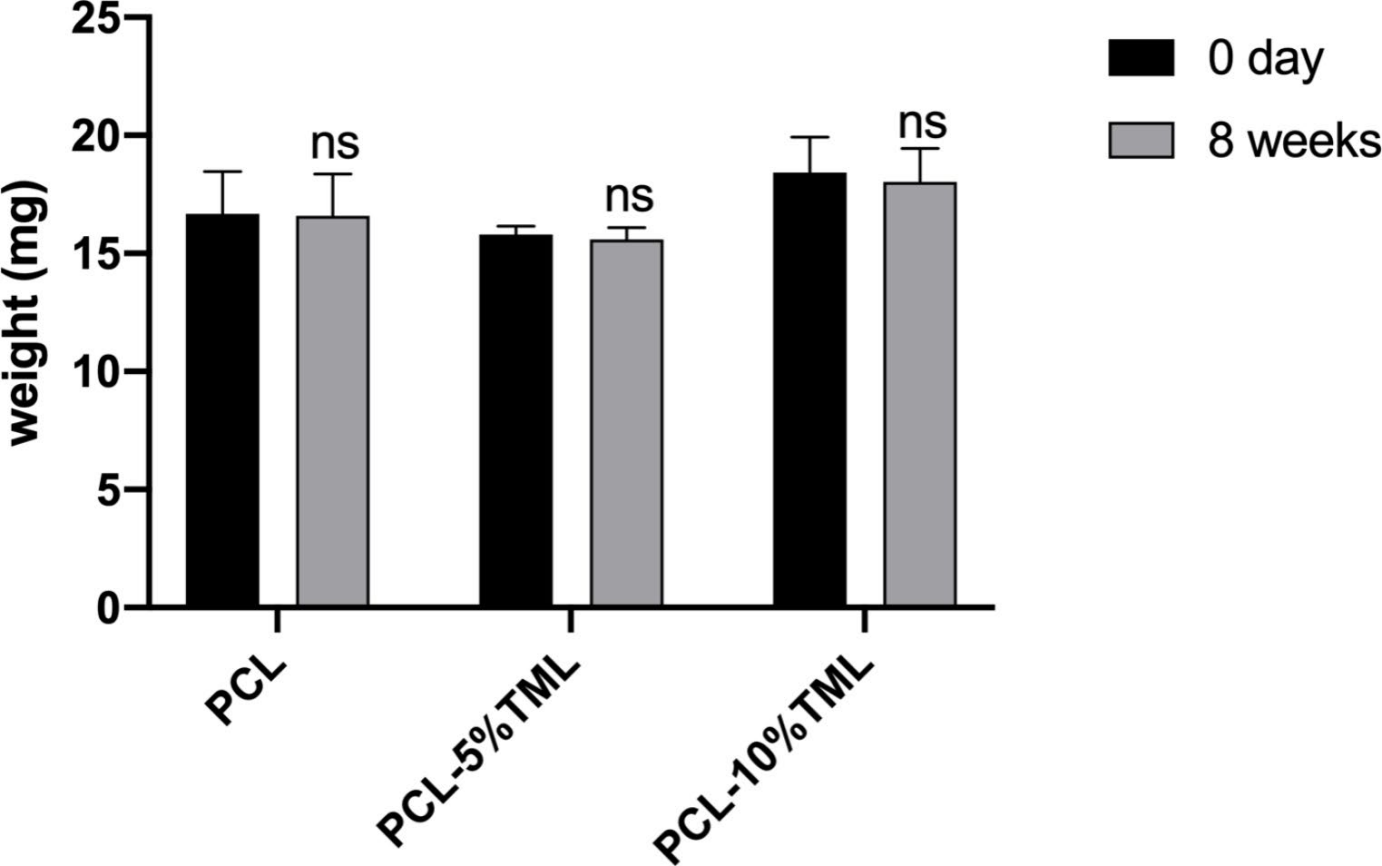
Change in weight of the PCL, PCL-5%TML and PCL-10%TML implants before and after the drug release experiment. Results represent mean ± SD, *N* = 3. ns, not significant

A drug release study was carried out to determine whether TML could be released from the implants. A gradual increase of TML release over 8 weeks in both the 5% and 10% formulations was observed (Fig. 6). The PCL-10%TML implant eluted the drugs at a higher concentration than the PCL-5%TML implant. By the end of week 1, the released drug from 1 mg TML implants were 3.39 µg (PCL-5%TML) and 7.42 µg (PCL-10%TML), respectively. Increasing the TML concentration by 5% in the implant resulted in a 118.9% increase in the drug released at the 1-week time point. By the end of week 8, the drug released from 1 mg PCL-5%TML and 1 mg PCL-10%TML implants were 7.74 µg per mg implant and 17.15 per mg implant, respectively, resulting in an increase of 121.6% in the 10% TML formulation compared to the 5% formulation at the 8-week time point. PCL is a well-known polymer with a very slow degradation rate due to its semi-crystalline nature [20]. After incubation in PBS for 8 weeks for the drug release experiment, all implants showed a small decrease in weight loss of 0.387% (PCL), 1.278% (PCL-5% TML) and 2.154% (PCL-10% TML), which is negligible (Fig. 7).

### Biocompatibility evaluation of the implants

**Fig. 8:**
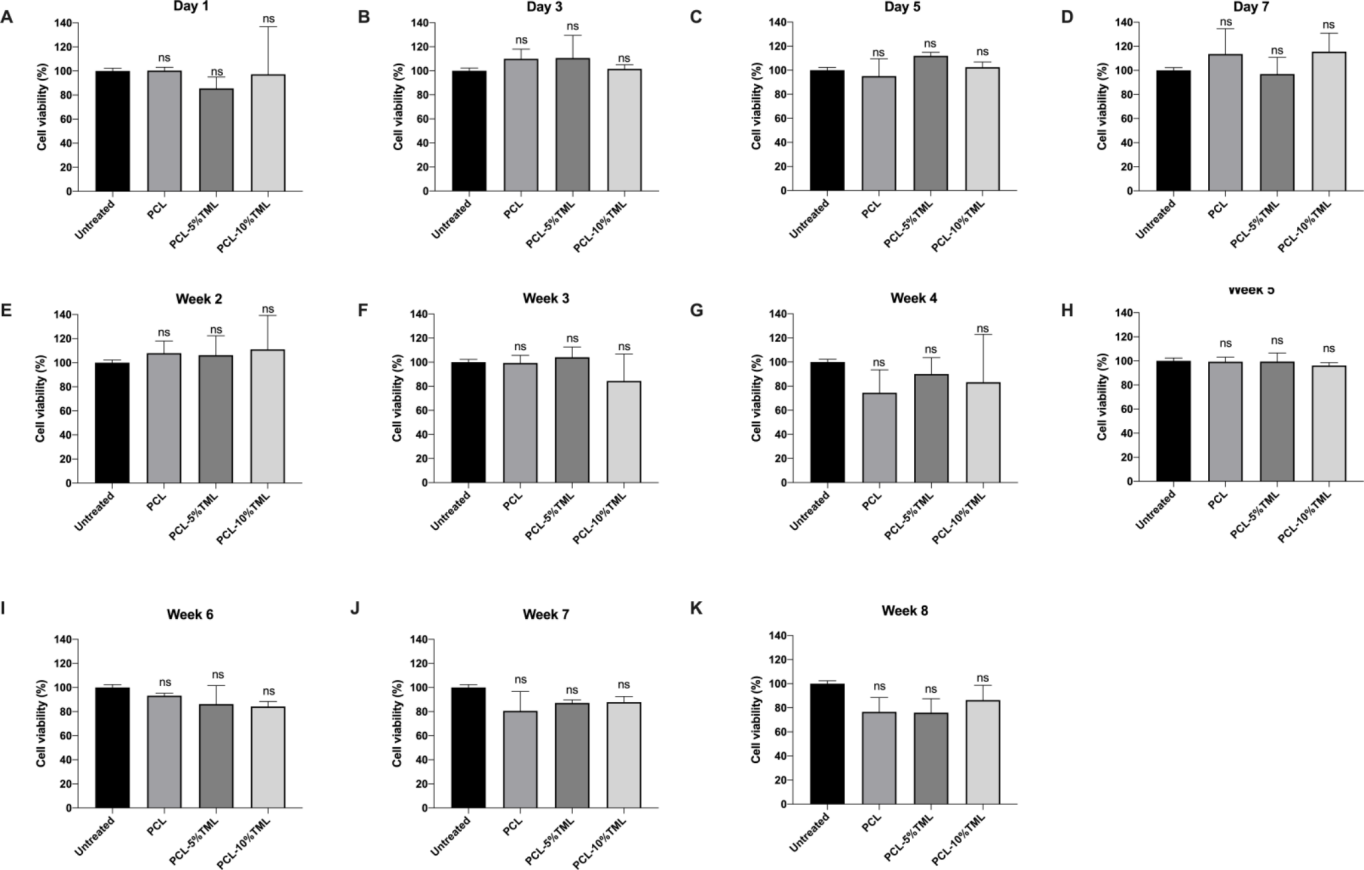
Cell viability of human trabecular meshwork cells after 1-day treatment with the drug solutions of the PCL, PCL-5%TML and PCL-10%TML implants collected from (**A**) Day 1, (**B**) Day 3, (**C**) Day 5, (**D**) Day 7, (**E**) Week 2, (**F**) Week 3, (**G**) Week 4, (**H**) Week 5, (**I**) Week 6, (**J**) Week 7, (**K**) Week 8. The cell viability was normalised against untreated cells. Results represent mean ± SD, *N* = 3. ns, not significant

To assess the safety of the implants, cell viability assays were carried out with drug solutions collected from PCL, PCL-5%TML, and PCL-10%TML implants at different time points. The values were normalised against untreated control cells (Fig. 8). At day 1, day 3, day 5, day 7, week 2, week 3, week 4, week 5, week 6, week 7 and week 8, all drug solutions collected from the three implants showed no statistically significant differences in human TM cells compared to the untreated cells. These findings demonstrate that PCL has good biocompatibility and is suitable for implantable applications.

## Discussion

Sustained DDSs provide a means to overcome the ubiquitous patient non-compliance, improper administration and chronic side effects associated with daily eye drops. 3DP technology have significant advantage over conventional for implant fabrication, such as hotmelt extrusion (HME) or extruded filament from the printer since 3DP offers unique benefits in terms of precision, customization, flexibility, and efficiency, making it an attractive option for pharmaceutical manufacturing [30–33]. PCL, known for its biocompatible and biodegradable nature, was used to print the implants. Another study that used PCL for 3DP DDS has shown that the polymer has good mechanical stability, thus making it ideal for ocular delivery [20].

Administration of TML eye drops is one of the most widely used medical treatments in glaucoma [34]. However, due to the low drug bioavailability and patient compliance issues [35], the majority of previous studies in the field of sustained TML administration focused on delivery platforms encompassing contact lenses and intracameral inserts [36, 37]. Nevertheless, these endeavours have been accompanied by notable limitations. For example, the critical properties of the contact lens, such as oxygen permeability and elastic modulus, restrict its application [38]. By co-delivering TML and hyaluronic acid, Desai et al., successfully developed a contact lens that can provide comfort to patient’s eyes; however, the temporal scope of TML release was confined to only 96 h, requiring frequent replacements to sustain therapeutic levels [39].

Samy et al., designed an intracameral insert that achieved zero-order release of TML for 3 months; however, the V-shaped configuration of the insert yielded unintended complications, including corneal epithelial and iris damage, as observed during the experimental phase on rabbit models [40]. Furthermore, the multilayer biodegradable implant incorporating TML, as developed by Ng et al., demonstrates a sustained drug-release profile capable of delivering therapeutic doses for up to three months in vitro. However, it necessitates a cumbersome preparation process [35]. Hence, to manufacture an intracameral implant for the sustained release of TML with a simple and highly reproducible method as well as to enable clinical translation, we developed an extended ophthalmic DDS with a miniaturised structure by 3DP technology. In this study, two concentrations of TML (5% and 10%) were used when printing the implants. Using these concentrations, it was possible to determine the optimal printing parameters by studying differences in the thermal, chemical, and structural behaviours of the implants. TML melts at a temperature of 202 °C to 203 °C [41] and PCL melts at a temperature of 60 °C [20]. Therefore, when printing at 150 °C, it is possible to conclude that there were no chemical interactions between the drug and polymer mix. When mixed with an active pharmaceutical ingredient (API), a higher pressure was used compared to PCL alone, which is the result of the higher viscosity when the polymer and drugs are mixed together. A previously established study correlates with these findings [42].

In the 3DP process, the application of heat during this procedure results in the alignment of polymer chains, thereby augmenting the level of crystallinity and subsequently mitigating surface cracks in the implants [43]. Like other 3DP implants, the printed implants had a smooth surface. The polymer mix melting efficiency was visualised by SEM imaging. TML implants showed a white powder-like residue that is likely due to TML agglomerations.

The chemical structure of the implant was further studied using FTIR to determine whether the manufacturing process caused any unwanted chemical interactions. A small difference was observed where a possible NH bond may be; however, this may be due to the presence of TML and not due to unwanted bonding [41]. A previous study using levofloxacin, also reported no observable differences at concentrations of 0.5%, however, as it increased to 1.5% a slight difference was noted corresponding to the drug [44]. This is interesting as the PCL-10%TML implant in this study showed no significant difference when compared with the PCL only implant.

In addition, DSC and TGA analysis were carried out to determine the thermal properties of printed implants as well as the pure powders. The reaction of the implants and powders to thermal stress was determined by DSC. The endothermic peaks of all implants were similar, at 59 °C to 63 °C. This is expected as most of the implant was composed of PCL. Crystallisation was seen in the powders and the PCL only implants. However, the TML-containing implants did not exhibit this, which is perhaps because PCL acted as a thermoprotectant. Despite the low melting temperatures, TGA analysis indicated that degradation does not occur at the 150 °C temperature at which printing was conducted.

An in vitro drug release assay was conducted to ensure that the implant was able to release the drug. The PCL-5%TML implant showed a gradual increase of released drug across the 8 weeks with a slight indication to a plateau seen towards the end of the study. The release from the PCL-10%TML implant was still increasing at the 56-day mark which was expected due to the higher concentration of TML. The time at which sustained release was achieved was not seen during the first 8 weeks of the drug release. Comparable findings were observed in a prior investigation by Ioannou et al., where PCL-chitosan implants loaded with 1% 5-FU by 3DP technology, achieved an extended sustained release duration with the capability to release 3.07 µg/mL of 5-fluorouracil at the end of week 8 [17]. In contrast, Di Luca and coworkers utilised 3DP to create a PCL-chitosan implant loaded with 1% 5-FU, but in order to serve a specialised function after the radiation therapy, this implant incorporated an additional PCL-based gold nanoparticle layer. This unique feature led to a distinct release profile, with a plateau reached at the 4-week mark and a cumulative release of 0.13 mg of 5-FU [20]. Furthermore, in consistence to the previous research [17], the degradation rates of the implants were very slow; this could be explained by the highly hydrophobic property and semi-crystalline nature of the PCL.

Ozurdex, an intracameral implant formulated using poly(lactic-co-glycolic acid) (PLGA) through a hot-melt extrusion (HME) process, has garnered substantial clinical utilisation as a sustained-release vehicle for dexamethasone [45]. In contrast to the drug elution kinetics observed with the PCL-TML implant configuration, recent publication data underline some distinctive features of Ozurdex’s drug release profile. Notably, Ozurdex only exhibited a limited burst release (∼ 10%) during the first week, followed by a comprehensive drug release phase spanning from weeks 3 to 4 [12]. This distinct behaviour stems from the intrinsic attributes of the loaded drug, dexamethasone, characterised by limited aqueous solubility (∼ 90 µg/mL) [12]. Consequently, only a small fraction of the drug, residing proximally to the implant’s surface, dissolves within the phosphate-buffered saline during the preliminary stages. Then subsequent autocatalytic hydrolysis of the PLGA matrix effectively alleviates the restraint on dexamethasone, expediting the overall drug release kinetics afterwards [46].

The safety assessment of the implants is critical for the long-term success of the implant incorporation and previous work has demonstrated the good biocompatibility of the PCL implants [17]. In this study, by incubating with drug solutions obtained from all three implants at various time points, the human TM cell viability exhibited no significant difference when compared to untreated cells. This result underscores the potential safety and biocompatibility of 3DP PCL implant formulations for sustained timolol release applications.

## Conclusions and future directions

In the current study, the printing parameters for Timolol containing-PCL based implants were established. The physicochemical characteristics as well as the drug-release kinetics of the prepared implants were investigated. This work demonstrated that 3DP can be used to fabricate PCL implants loaded with TML, which can deliver the target drug at a therapeutic dose for 8 weeks. The study acts as a proof of concept with regards to the development of various DDSs for glaucoma therapies. However, to pave the way for transferring the resulting DDS to a clinical setup there is a need for investigating the physical stability of the 3DP DDS in a quantifiable manner using in vivo models to validate the drug release from the implants in a biological environment.

## Data Availability

The datasets generated during and/or analysed during the current study are available from the corresponding author on reasonable request.

## References

[1] Tezel G. Multifactorial Pathogenic Processes of Retinal Ganglion Cell Degeneration in Glaucoma towards Multi-Target Strategies for Broader Treatment Effects. Cells. Jun 2 2021:10(6). 10.3390/cells10061372. 10.3390/cells10061372 PMC8228726 34199494

[2] Allison K, Patel D, Alabi O. Epidemiology of Glaucoma: The Past, Present, and Predictions for the Future. Cureus. Nov 24 2020; 12(11):e11686. 10.7759/cureus.11686. 10.7759/cureus.11686 PMC7769798 33391921

[3] Kang JM, Tanna AP. Glaucoma. Med Clin North Am. May 2021;105(3):493–510. 10.1016/j.mcna.2021.01.004. 10.1016/j.mcna.2021.01.004 33926643

[4] Qin M, Yu-Wai-Man C. Glaucoma: novel antifibrotic therapeutics for the trabecular meshwork. Eur J Pharmacol. Sep 5 2023;954:175882. 10.1016/j.ejphar.2023.175882. 10.1016/j.ejphar.2023.175882 PMC10804937 37391006

[5] Sapowadia A et al. Biomaterial Drug Delivery Systems for Prominent Ocular Diseases. Pharmaceutics. Jul 15. 2023;15(7)10.3390/pharmaceutics15071959. 10.3390/pharmaceutics15071959 PMC10383518 37514145

[6] Naik S, Pandey A, Lewis SA, Rao BSS, Mutalik S. Neuroprotection: A versatile approach to combat glaucoma, Eur J Pharmacol. Aug 15 2020;881:173208. 10.1016/j.ejphar.2020.173208. 10.1016/j.ejphar.2020.173208 32464192

[7] Sah AK, Suresh PK. Medical management of glaucoma: focus on ophthalmologic drug delivery systems of timolol maleate. Artif Cells Nanomed Biotechnol. May 2017;45(3):448–59. 10.3109/21691401.2016.1160917. 10.3109/21691401.2016.1160917 27002850

[8] Tagalakis AD, et al. In vitro and in vivo delivery of a sustained release nanocarrier-based formulation of an MRTF/SRF inhibitor in conjunctival fibrosis. J Nanobiotechnol. Nov 27 2018;16(1):97. 10.1186/s12951-018-0425-3. 10.1186/s12951-018-0425-3 PMC6258153 30482196

[9] Miller PE, Eaton JS. Medical anti-glaucoma therapy: Beyond the drop, (in eng). Vet Ophthalmol. Mar 2021;24(Suppl 1):2–15. 10.1111/vop.12843. 10.1111/vop.12843 33164328

[10] Sirinek PE, Lin MM. Intracameral sustained release bimatoprost implants (Durysta). Semin Ophthalmol. Apr 3 2022;37(3):385–390. 10.1080/08820538.2021.1985145. 10.1080/08820538.2021.1985145 34586961

[11] Cao Y, Samy KE, Bernards DA, Desai TA. Recent advances in intraocular sustained-release drug delivery devices. Drug Discov Today. Aug 2019;24(8):1694–1700. 10.1016/j.drudis.2019.05.031. 10.1016/j.drudis.2019.05.031 PMC6708500 31173915

[12] Costello MA, et al. Reverse engineering the Ozurdex dexamethasone intravitreal implant. Int J Pharm. Mar 5 2023;634:122625. 10.1016/j.ijpharm.2023.122625. 10.1016/j.ijpharm.2023.122625 36690129

[13] Bom S, Martins AM, Ribeiro HM, Marto J. Diving into 3D (bio)printing: A revolutionary tool to customize the production of drug and cell-based systems for skin delivery, Int J Pharm. Aug 10 2021;605:120794. 10.1016/j.ijpharm.2021.120794. 10.1016/j.ijpharm.2021.120794 34119578

[14] Leng S. Anatomic modeling using 3D printing: quality assurance and optimization. 3D Printing in Med. 2017;3(1): 6. 2017/04/26 10.1186/s41205-017-0014-3 10.1186/s41205-017-0014-3 PMC5954797 29782614

[15] Fakhoury Y, Ellabban A, Attia U, Sallam A, Elsherbiny S. Three-dimensional printing in ophthalmology and eye care: current applications and future developments. Ther Adv Ophthalmol. Jan-Dec 2022;14:25158414221106682. 10.1177/25158414221106682. 10.1177/25158414221106682 PMC9247992 35782482

[16] Lamprou DA, Douroumis D, Andrews GP, Jones DS. 3D printing in pharmacological and pharmaceutical sciences. J Pharm Pharmacol. Oct 10 2022;74(10):1365–6. 10.1093/jpp/rgac049. 10.1093/jpp/rgac049 36062934

[17] Ioannou N et al. 3D-printed long-acting 5-fluorouracil implant to prevent conjunctival fibrosis in glaucoma. J Pharm Pharmacol, 75, 2, pp. 276–86, Feb 8 2023, 10.1093/jpp/rgac100. 10.1093/jpp/rgac100 PMC10813237 36617180

[18] Mohamdeen YMG, et al. Development of 3D printed drug-eluting contact lenses. J Pharm Pharmacol. Oct 10 2022;74(10):1467–76. 10.1093/jpp/rgab173. 10.1093/jpp/rgab173 34928372

[19] Ding J, Zheng H, Ji X. One-step side-by-side 3D printing constructing linear full batteries. Chem Commun (Camb), 58, 34, pp. 5241–4, Apr 26 2022, 10.1039/d2cc00915c. 10.1039/d2cc00915c 35388828

[20] Di Luca M, et al. 3D printed biodegradable multifunctional implants for effective breast cancer treatment. Int J Pharm. Nov 3 2022;629:122363. 10.1016/j.ijpharm.2022.122363. 10.1016/j.ijpharm.2022.122363 36336202

[21] Corduas F, Mathew E, McGlynn R, Mariotti D, Lamprou DA, Mancuso E. Melt-extrusion 3D printing of resorbable levofloxacin-loaded meshes: emerging strategy for urogynaecological applications. Mater Sci Eng C Mater Biol Appl. Dec 2021;131:112523. 10.1016/j.msec.2021.112523. 10.1016/j.msec.2021.112523 34857302

[22] Lance KD, et al. In vivo and in vitro sustained release of ranibizumab from a nanoporous thin-film device. Drug Deliv Transl Res. Dec 2016;6(6):771–80. 10.1007/s13346-016-0298-7. 10.1007/s13346-016-0298-7 PMC5097678 27178165

[23] Won JY et al. 3D printing of drug-loaded multi-shell rods for local delivery of bevacizumab and dexamethasone: a synergetic therapy for retinal vascular diseases. Acta Biomater, 116, pp. 174–85, Oct 15 2020, 10.1016/j.actbio.2020.09.015. 10.1016/j.actbio.2020.09.015 32927088

[24] Hu T, et al. Design, preparation and performance of a novel drug-eluting stent with multiple layer coatings. Biomater Sci. Aug 22 2017;5(9):1845–57. 10.1039/c7bm00417f. 10.1039/c7bm00417f 28676873

[25] Hafez HM, Elshanawany AA, Abdelaziz LM, Mohram MS. Design of experiment (DOE) utilization to develop a simple and robust RP-UPLC technique for Stability Indicating Method of Ciprofloxacin Hydrochloride and Metronidazole in tablets. Eurasian J Anal Chem. 2015;10(2):84–105.

[26] Luo J et al. Non-viral gene therapy in trabecular meshwork cells to prevent fibrosis in minimally invasive glaucoma surgery, *Pharmaceutics*, vol. 14, no. 11, pp. 2472–2488, 2022, 10.3390/pharmaceutics14112472. 10.3390/pharmaceutics14112472 PMC9693853 36432663

[27] Domínguez-Robles J, et al. Poly(caprolactone)/lignin-based 3D-printed dressings loaded with a novel combination of bioactive agents for wound-healing applications. Sustainable Mater Technol. 2023;35. 10.1016/j.susmat.2023.e00581.

[28] Dominguez-Robles J, et al. Development of drug loaded cardiovascular prosthesis for thrombosis prevention using 3D printing. Mater Sci Eng C Mater Biol Appl. Oct 2021;129:112375. 10.1016/j.msec.2021.112375. 10.1016/j.msec.2021.112375 PMC8505756 34579894

[29] Domínguez-Robles J et al. Use of 3D Printing for the Development of Biodegradable Antiplatelet Materials for Cardiovascular Applications, (in eng), *Pharmaceuticals (Basel)*, vol. 14, no. 9, Sep 11., 2021, 10.3390/ph14090921. 10.3390/ph14090921 PMC8466262 34577621

[30] Thakkar V. Formulation and in vitro - in vivo evaluations of Timolol maleate viscous eye drops for the treatment of glaucoma. Eur J Biomedical Pharm Sci. 2016;3(9):573–85.

[31] Zhang J, Wehrle E, Rubert M, Muller R. 3D Bioprinting of Human Tissues: Biofabrication, Bioinks, and Bioreactors. Int J Mol Sci. Apr 12 2021;22(8). 10.3390/ijms22083971. 10.3390/ijms22083971 PMC8069718 33921417

[32] Kotta S, Nair A, Alsabeelah N. 3D Printing Technology in Drug Delivery: recent progress and application. Curr Pharm Des. 2018;24(42):5039–48. 10.2174/1381612825666181206123828. 30520368 10.2174/1381612825666181206123828

[33] Warsi MH, Yusuf M, Al Robaian M, Khan M, Muheem A, Khan S. 3D Printing methods for Pharmaceutical Manufacturing: Opportunity and challenges. Curr Pharm Des. 2018;24(42):4949–56. 10.2174/1381612825666181206121701. 30520367 10.2174/1381612825666181206121701

[34] Stewart WC, Stewart JA, Mychaskiw MA. Cost-effectiveness of latanoprost and timolol maleate for the treatment of glaucoma in Scandinavia and the United Kingdom, using a decision-analytic health economic model. Eye (Lond). Jan 2009;23(1):132–40. 10.1038/sj.eye.6702964. 10.1038/sj.eye.6702964 17721497

[35] Ng XW, Liu KL, Veluchamy AB, Lwin NC, Wong TT, Venkatraman SS. A biodegradable ocular implant for long-term suppression of intraocular pressure. Drug Deliv Transl Res. Oct 2015;5(5):469–79. 10.1007/s13346-015-0240-4. 10.1007/s13346-015-0240-4 PMC4551556 26100093

[36] Hiratani H, Alvarez-Lorenzo C. Timolol uptake and release by imprinted soft contact lenses made of N,N-diethylacrylamide and methacrylic acid. J Control Release. Oct 4 2002;83(2):223– 30. 10.1016/s0168-3659(02)00213-4. 10.1016/s0168-3659(02)00213-4 12363448

[37] Schultz CL, Poling TR, Mint JO. A medical device/drug delivery system for treatment of glaucoma. Clin Exp Optom. Jul 2009;92(4):343–8. 10.1111/j.1444-0938.2009.00370.x. 10.1111/j.1444-0938.2009.00370.x 19389129

[38] Jung HJ, Abou-Jaoude M, Carbia BE, Plummer C, Chauhan A. Glaucoma therapy by extended release of timolol from nanoparticle loaded silicone-hydrogel contact lenses. J Control Release. Jan 10 2013;165(1):82– 9. 10.1016/j.jconrel.2012.10.010. 10.1016/j.jconrel.2012.10.010 23123188

[39] Desai AR, et al. Co-delivery of timolol and hyaluronic acid from semi-circular ring-implanted contact lenses for the treatment of glaucoma: in vitro and in vivo evaluation. Biomater Sci. May 29 2018;6(6):1580–91. 10.1039/c8bm00212f. 10.1039/c8bm00212f 29708242

[40] Samy KE, et al. Co-delivery of Timolol and Brimonidine with a Polymer Thin-Film intraocular device. J Ocul Pharmacol Ther. Mar 2019;35(2):124–31. 10.1089/jop.2018.0096. 10.1089/jop.2018.0096 PMC6450452 30615539

[41] Dipika Chavda VT, Soni T, Gandhi T. Formulation and in vitro - in vivo evaluations of Timolol maleate visous eye drops for the treatment of glaucoma. European J Biomed Pharmaceutical Sci. 2016;3(9):573–585. [Online]. Available: https://www.ejbps.com/ejbps/abstract_id/1718.

[42] Hall Barrientos IJ et al. Fabrication and characterisation of drug-loaded electrospun polymeric nanofibers for controlled release in hernia repair. Int J Pharm. Jan 30. 2017;517(1–2):329–337. 10.1016/j.ijpharm.2016.12.022. 10.1016/j.ijpharm.2016.12.022 27988377

[43] Fialho SL, da Silva Cunha A. Manufacturing techniques of biodegradable implants intended for intraocular application. Drug Deliv. 2005;12(2):109– 16. 10.1080/10717540590921432. 10.1080/10717540590921432 15824036

[44] Glover K, Mathew E, Pitzanti G, Magee E, Lamprou DA. 3D bioprinted scaffolds for diabetic wound-healing applications. Drug Deliv Transl Res. Aug 2023;13(8):2096–2109. 10.1007/s13346-022-01115-8. 10.1007/s13346-022-01115-8 PMC10315349 35018558

[45] Kuo HK, Chen YH, Wu PC, Kuo YH. The Effects of Ozurdex(R) (Dexamethasone Intravitreal Implant) on Experimental Proliferative Vitreoretinopathy. Ophthalmologica. 2015;233(3–4):198–203. 10.1159/000371901. 10.1159/000371901 25721986

[46] Tamani F, et al. Mechanistic explanation of the (up to) 3 release phases of PLGA microparticles: Diprophylline dispersions. Int J Pharm. Dec 15 2019;572:118819. 10.1016/j.ijpharm.2019.118819. 10.1016/j.ijpharm.2019.118819 31726196

